# Bezoars-Induced Small Bowel Obstructions due to Dried Persimmons that Required Multiple Surgeries: A Case Report

**DOI:** 10.70352/scrj.cr.26-0419

**Published:** 2026-10-31

**Authors:** Yoshikuni Yonenaga, Ryota Ito

**Affiliations:** 1 Department of Surgery, Ako City Hospital, Ako, Hyogo, Japan; 2 Department of Surgery, Nishiwaki Municipal Hospital, Nishiwaki, Hyogo, Japan

**Keywords:** diospyrobezoar, dried persimmon, intestinal obstruction, perforation

## Abstract

**INTRODUCTION:**

A bezoar is an indigestible conglomeration trapped in the gastrointestinal tract. Persimmon phytobezoars, known as diospyrobezoars, are formed after the frequent consumption of persimmons and are a distinct type of phytobezoar characterized by their hard consistency.

**CASE PRESENTATION:**

A 74-year-old female presented to our hospital with persistent abdominal pain and vomiting, and an intestinal strangulating obstruction was suspected. An emergency operation was performed, and a dietary small bowel obstruction was diagnosed. A small intestinal resection, including removal of a foreign body resembling a sponge-like mass, was performed. During detailed questioning, we asked her about any history of persimmon ingestion, but she denied it. The symptoms recurred on POD 8, and another dietary small bowel obstruction was suspected after a CT examination. Conservative treatment, including Coca-Cola dissolution therapy, was performed, but the symptoms were not alleviated, and she developed a fever on POD 24. On POD 30—the 22nd day after the 2nd onset—she suddenly remembered that she had eaten 16 dried persimmons between 24 and 19 days before the onset of symptoms. A 2nd operation was performed the following day. Because feces were found in the abdominal cavity and the perforated site could not be found laparoscopically, we converted to laparotomy. At the location of the obstruction in the ileum, a foreign body and a perforation were seen, and a partial ileectomy was performed. The foreign body also resembled a sponge-like mass, and a component analysis showed that it consisted of 98% tannin. A diospyrobezoar was finally diagnosed.

**CONCLUSIONS:**

Because multiple diospyrobezoars could exist in the gastrointestinal tract, we should check for additional bezoars throughout the entire tract. Much more detailed questionnaires, including both dried and ripe persimmons, are needed. During conservative therapy, the occurrence of severe complications should be recognized.

## Abbreviations

SBFS
: small bowel feces sign

## INTRODUCTION

Bezoars are an indigestible conglomeration trapped in the gastrointestinal tract and are responsible for 0.4%–4% of cases of mechanical intestinal obstruction.^1)^ They are mainly classified into 4 types depending on the material constituting their indigestible mass: plant materials such as fibers, skins, and seeds of vegetables and fruits (phytobezoars); ingested hair (trichobezoars); medications (pharmacobezoars); and milk protein in milk-fed infants (lactobezoars).^2)^ Of these 4 types, phytobezoars are the most common, and persimmons in particular are a representative cause. Persimmon phytobezoars, known as diospyrobezoars, are formed after the frequent consumption of persimmons and are a distinct type of phytobezoar characterized by their hard consistency.

Herein, we report a case of diospyrobezoars-induced small bowel obstructions that required reoperation.

## CASE PRESENTATION

In January 2023, a 74-year-old female presented with persistent abdominal pain and vomiting since the night before. A contrast-enhanced abdominal CT was performed, and our radiologist noted that intestinal dilation and a transition to a non-dilated intestine were present in the lower abdomen, and rotation of the mesenteric vessels was seen in that area, raising suspicion of volvulus (**Fig.1A** and **1B**). As a result, an intestinal strangulating obstruction was suspected. She was referred to our department with no history of abdominal surgeries. At this point, we could not recognize the presence of a gastric bezoar at the transition zone of the caliber change. We performed an emergency operation using a laparoscopic approach, during which we investigated from the ileum end, and a caliber change and a tumorous lesion were observed at a site approximately 30 cm from the terminal ileum (**Fig. 2A**). The lesion was extracted outside the abdominal cavity. On palpation, it was not hard and felt compressible, and a dietary small bowel obstruction was eventually diagnosed (**Fig. 2B**). A small intestinal resection including the foreign body was performed. The foreign body had a darkish-brown color on the surface layer (**Fig. 3A**), an orange color in the inner part, and it resembled a sponge-like mass (**Fig. 3B**). We retrospectively reviewed the preoperative contrast-enhanced CT and found a low-density mass with bubbles inside that was impacted on the anal side of the dilated small intestine (**Fig. 1C** and **1D**). As for the causative food, she had ingested freeze-dried tofu (koya-tofu) and bean snacks the day before the surgery. Other than these, she had most recently ingested rice cakes (kagamimochi). At the same time, we inquired about any history of persimmon ingestion, but she denied it. We speculated that freeze-dried tofu was the most likely candidate because of the spongy texture of the mass. The patient resumed oral intake after surgery, but the intestinal obstruction recurred on POD 8. A low-density mass with bubbles inside was found at the transition zone of the intestinal obstruction on plain CT examinations. A nasogastric tube was then inserted into the stomach. CT findings after the upper gastrointestinal series on POD 9 also showed that the low-density mass with bubbles inside was impacted on the anal side of the dilated small intestine (**Fig. 4**). We retrospectively reviewed the preoperative contrast-enhanced CT again and found a low-density mass in the stomach (**Fig. 1E** and **1F**). We supposed that the residual bezoar had migrated into the small intestine and eventually caused a mechanical obstruction. This time, we decided on conservative treatment with the hope that the obstructing mass would migrate to the colon. The placement of a long intestinal tube into the small intestine did not improve the disease conditions, so dissolution therapy with Coca-Cola infusion was initiated. One hundred milliliters of Coca-Cola was infused through a long intestinal tube, with a 1-hour clamp of the tube, every 2 hours, 5 times a day.^3)^ This therapy lasted 6 days and started on the 8th day after the recurrence. There was a small reduction in the size of the mass, and though there was a slight migration, it did not reach the colon. As the discharge of intestinal fluids had decreased, the long intestinal tube was removed on the 14th day, but the small bowel obstruction relapsed and the tube was reinserted on the 17th day. On the other hand, as a fever of 38.3°C and elevated C-reactive protein (CRP) levels (16.21 mg/dL) in a blood test were observed on the 16th day, cefmetazole was administered from the 16th to the 21st day (**Fig. 5**). Since the patient’s condition did not improve, an operation was scheduled for the 23rd day after the 2nd onset. While we were explaining the operation to obtain consent the day before, she suddenly recalled ingesting 2 skewers of dried persimmons (16 in total) around 24 to 19 days before the 1st onset of symptoms, as many as 3–4 per day. She had the 2nd laparoscopic operation on the 23rd day after the 2nd onset—the 31st day after the 1st operation. Although a small amount of feces was found in the abdominal cavity (**Fig. 6A**), the perforated site could not be found laparoscopically due to intraperitoneal adhesions. Thus, we converted to a laparotomy. The pelvic site of adhesion with a perforated area of small intestine was seen and separated (**Fig. 6B**), and a foreign body and a perforation were found at the location of the obstruction in the ileum (**Fig. 6C**). As a result, a partial ileectomy was performed. CT findings after the upper gastrointestinal series did not show any other low-density mass with bubbles inside the gastrointestinal tract. In addition, when we inserted a long intestinal tube using upper endoscopy, we checked for the absence of bezoars from the stomach to the 2nd portion of the duodenum. Furthermore, during the 2nd surgery, after we converted to laparotomy, we checked the stomach to the terminal end by palpation. The resected specimen showed necrosis associated with an ulcer (**Fig. 6D**). The foreign body resembled a sponge-like mass as earlier (**Fig. 6E**). A component analysis using infrared spectroscopy showed the body consisted of 98% tannin, and a diospyrobezoar was finally diagnosed. The patient did well in the postoperative period and was discharged 12 days after the 2nd operation. She did not experience any recurrence for 3 years and 2 months after the 2nd operation.

**Fig. 1:**
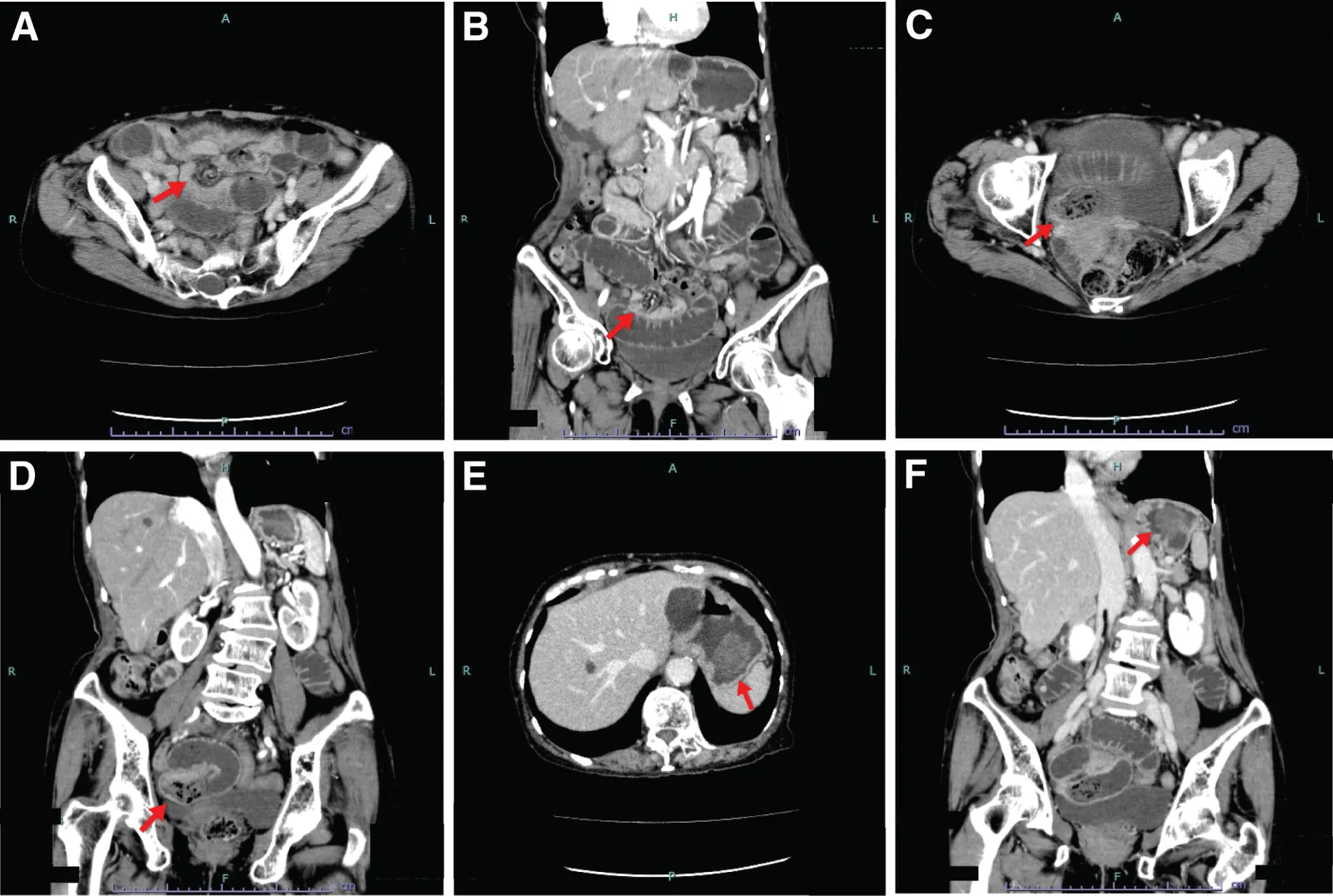
Preoperative contrast-enhanced CT findings. (**A**) Axial and (**B**) coronal CT findings showed that intestinal dilation and a transition to a non-dilated intestine were present in the lower abdomen, and rotation of the mesenteric vessels was suspected (arrows). (**C**) Axial and (**D**) coronal CT findings showed a low-density mass with bubbles inside that was impacted on the anal side of the dilated small intestine (arrows). (**E**) Axial and (**F**) coronal preoperative CT findings showed a low-density mass in the stomach (arrows).

**Fig. 2:**
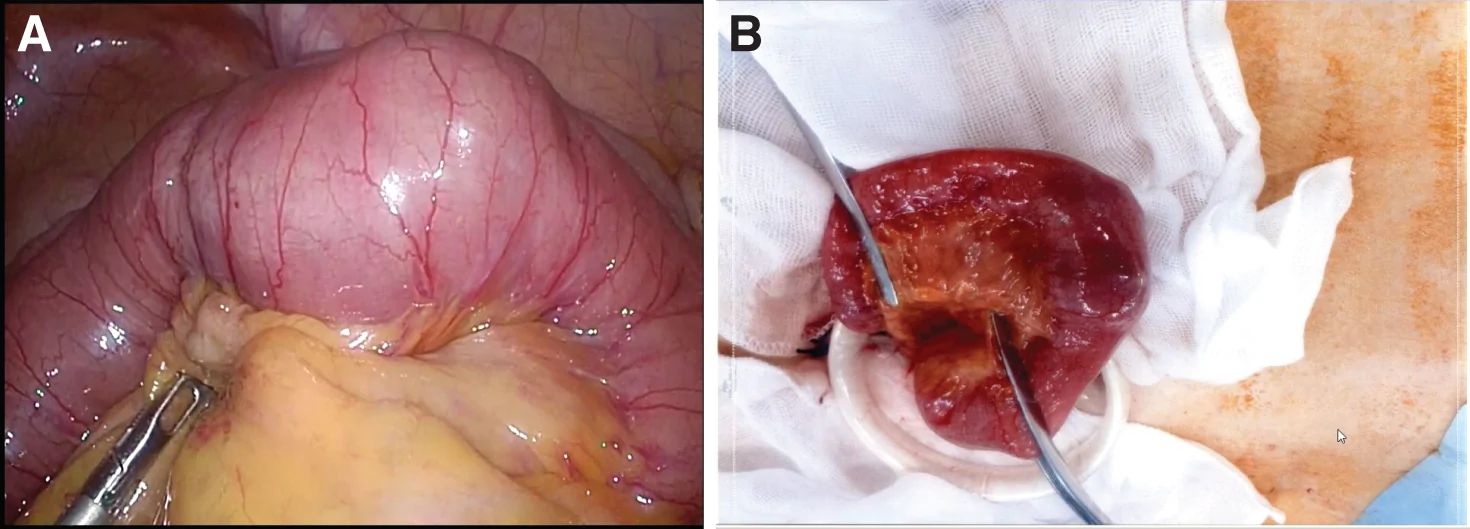
Intraoperative findings. (**A**) A tumorous lesion in the ileum was suspected. (**B**) A bolus of food was recognized by palpation.

**Fig. 3:**
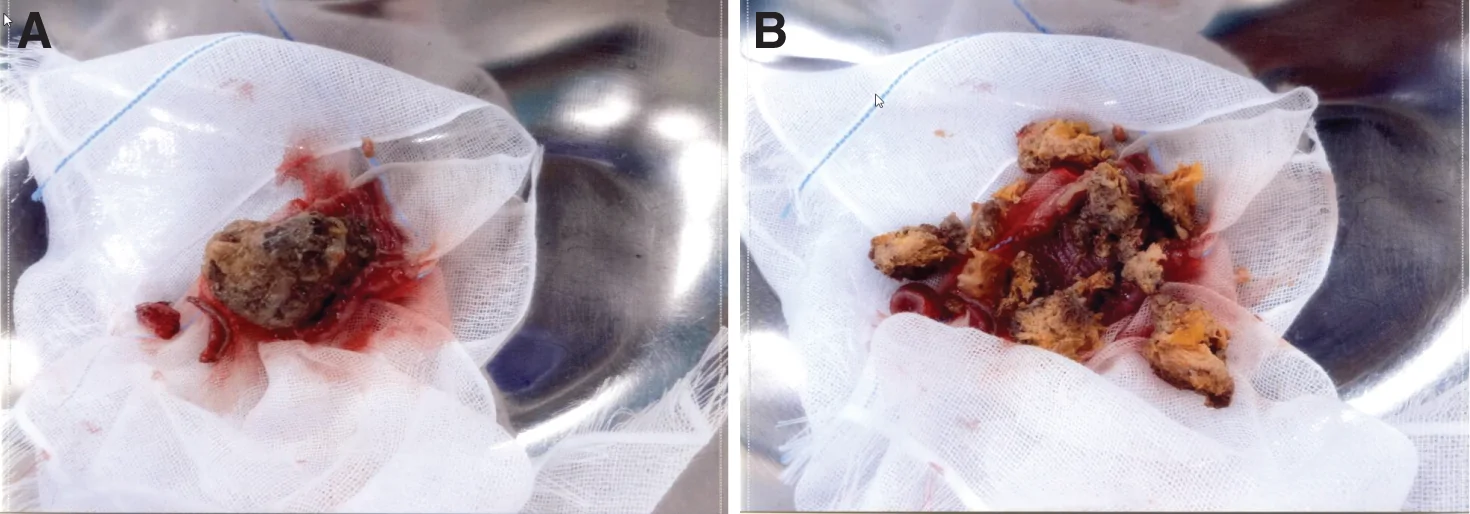
Findings showing the foreign body. (**A**) It had a darkish-brown color on the surface layer. (**B**) It had an orange color in the inner part, and pieces of it resembled a sponge-like mass.

**Fig. 4:**
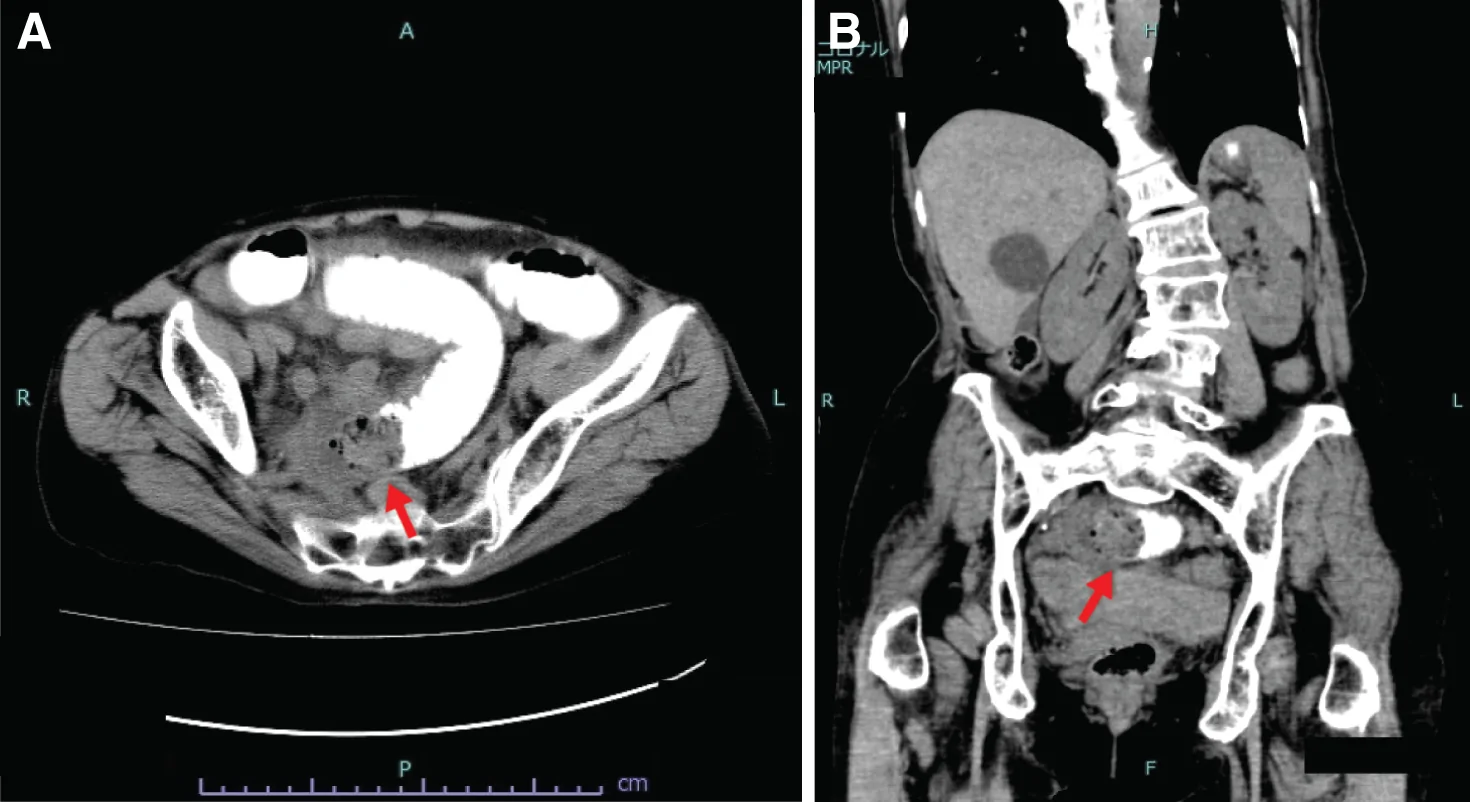
CT findings. (**A**) Axial and (**B**) coronal CT findings after the upper gastrointestinal series on POD 9. They showed a well-defined mass lesion with bubbles inside that was impacted on the anal side of the dilated small intestine (arrows).

**Fig. 5:**
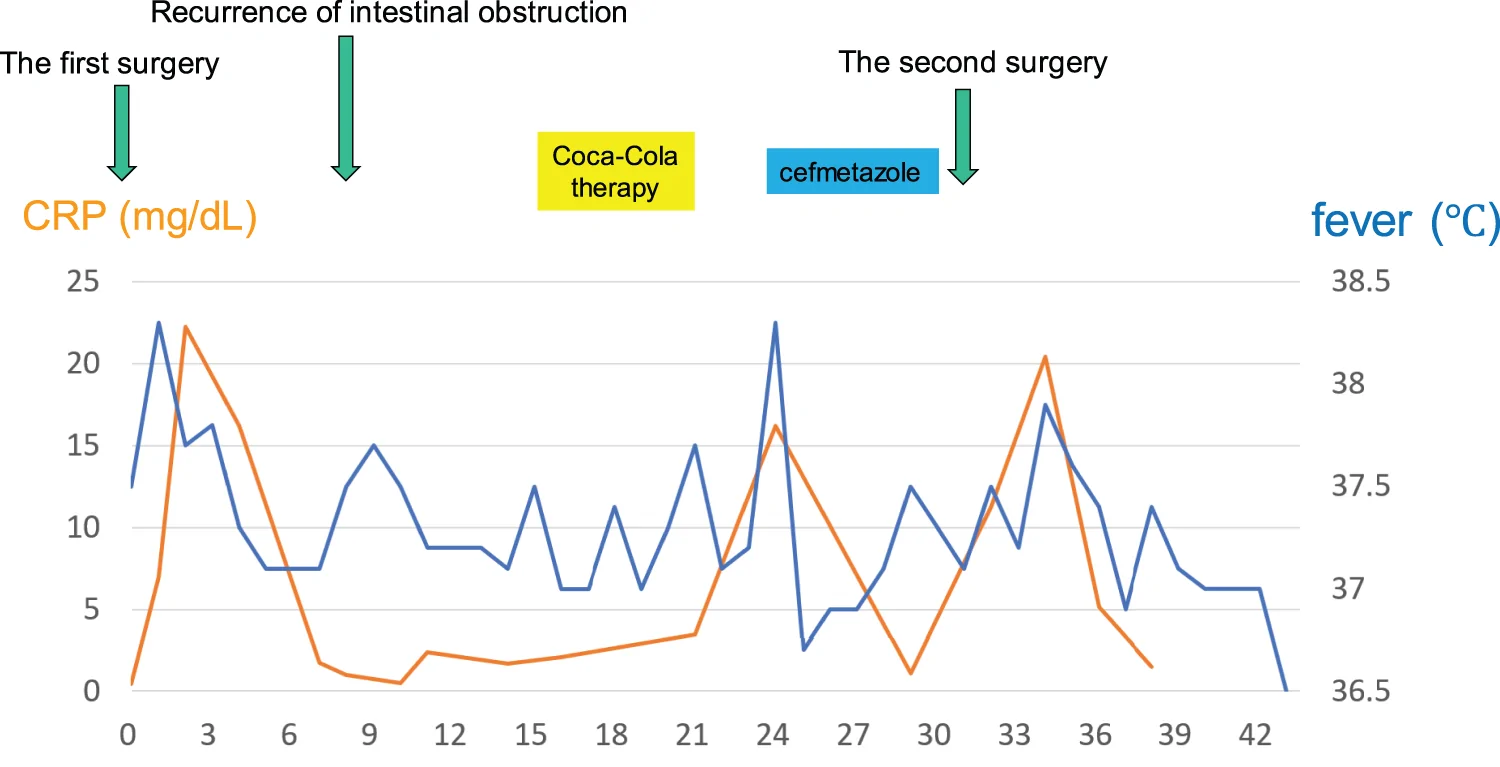
Clinical course of this case after the 1st surgery.

**Fig. 6:**
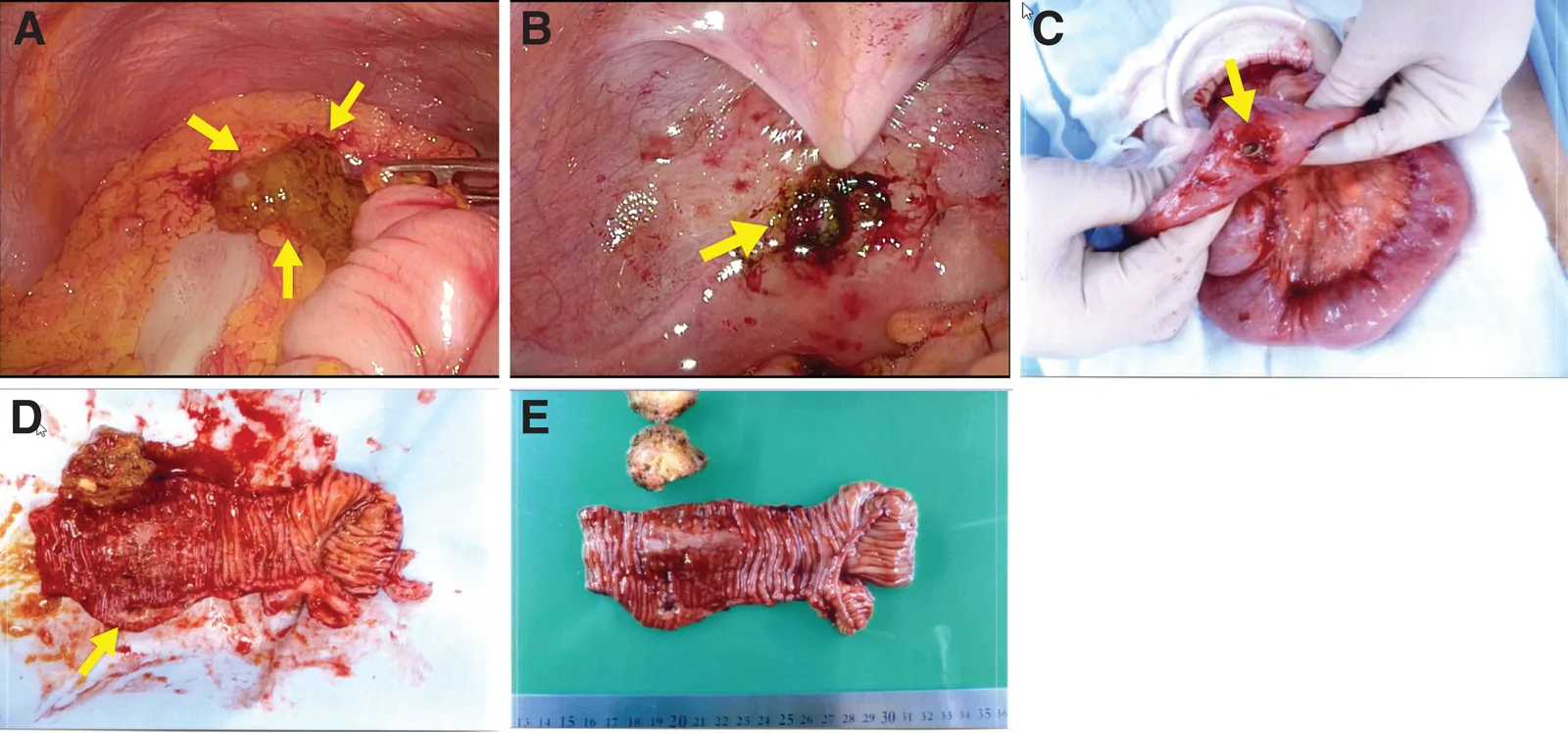
Intraoperative findings and the resected specimen. (**A**) A small amount of feces was found in the abdominal cavity (arrows). (**B**) The pelvic site of adhesion with a perforated area of small intestine was seen after the adhesiolysis (arrow). (**C**) A foreign body and a perforation were found at the location of the obstruction in the ileum (arrow). (**D**) The resected specimen showed necrosis associated with an ulcer (arrow). (**E**) The split specimen resembled a sponge-like mass.

## DISCUSSION

In this case, we learned many lessons. We were unable to make an accurate preoperative diagnosis and did not discover the patient’s history of dried persimmon ingestion. We performed a laparoscopic-assisted surgery, a procedure less commonly used in these cases. We encountered the recurrence of bezoars-induced small bowel obstructions, and the patient was resistant to Coca-Cola dissolution treatment. During conservative treatment, we discovered an intestinal perforation at the site where the bezoar was impacted. Among the aforementioned lessons, we identified the following 3 important clinical issues.

First, bezoars other than the one responsible for the intestinal obstruction might exist. Phytobezoars are usually visualized by CT as an ovoid or round mass occupying the gastrointestinal tract with air bubbles retained inside and a mottled appearance.^2)^ Kawano et al.^4)^ described a “bubbly mass and impaction” at the distal site of a small bowel dilatation, which is a characteristic sign of small bowel obstruction due to diet. Altintoprak et al.^5)^ reported that among 39 patients with bezoars that were surgically removed, 41.0% (16 patients) had multiple bezoar locations in the gastrointestinal tract, and the percentage of multiple bezoars located in the stomach and ileum, as in this case, was 12.8% (5 patients). Tsukioka et al.^6)^ reported intestinal obstructions due to 2 diospyrobezoars with reoperation and cited another case report in Japan. Hoover et al.^7)^ also emphasized this important detail in a report of a patient who required a 2nd operation because of an overlooked bezoar, despite preoperative CT examination. Because multiple diospyrobezoars could exist in the gastrointestinal tract, we should have searched for additional bezoars from the stomach to the ileum end during the 1st operation and reviewed whether other bezoars were present in the stomach on the preoperative CT examinations.^8)^ If we had detected residual bezoars in the stomach on the CT in the operating theater, they could have been removed by endoscopic procedures as soon as possible after the operation. Dikicier et al.^1)^ reported that endoscopic methods have become the main treatment for phytobezoars in the stomach. Ladas et al.^9)^ reported a very high success rate (84.6%) in gastric bezoars using a combination of endoscopic treatment and Coca-Cola treatment. Endo et al.^10)^ also reported that after the patient consumed Coca-Cola Zero for 4 days, the bezoars of the stomach became friable and were disintegrated using a polypectomy snare. Thus, early postoperative endoscopic removal, or combined with drinking Coca-Cola when necessary, would be feasible if we had detected residual bezoars in the stomach on the CT in the operating theater. Dikicier et al.^1)^ proposed in their review paper that the treatment of choice is surgical for gastric bezoars in which endoscopic treatment failed. In this case, we also thought that an endoscopic approach would be the best option as an initial treatment. Laparoscopic surgeries for bezoars are increasing,^1)^ and because it is usually more difficult to search for additional bezoars in the gastrointestinal tract than during open surgery, keeping in mind the possibility of residual bezoars, during laparoscopic surgery, the stomach and small intestine should be sequentially inspected with forceps.

Second, detailed and repeated inquiries about ingestion histories are needed. In this case, the patient did not associate dried persimmons with persimmons. It is therefore essential to ask the patient not only about the ingestion history of persimmons themselves but also about processed food products such as dried persimmons. In addition, long-term ingestion histories should also be collected. Actually, the patient ingested dried persimmons 24 to 19 days before the onset of the symptoms in this case. Dried persimmons are available in Japan in the winter and are also made in several countries in Asia. In Japan, the practice in which kagamimochi (mirror-shaped rice cakes) with skewered dried persimmons are displayed during the New Year’s Holidays and then eaten still exists. The most valuable method for determining the location and etiology of intestinal obstructions is contrast-enhanced CT.^1)^ However, in this case, we were unable to make a preoperative diagnosis of an intestinal obstruction due to bezoars on CT without information about the recent ingestion history of dried persimmons. SBFS can appear similar to a bezoar radiologically, that is, an isolated amorphous mass mottled with gas bubbles.^11)^ It is frequently found within the lumen of a relatively long segment of dilatated small bowel loop, and the incidence is approximately 8% in small bowel obstruction cases.^12)^ In this case, our radiologist might have erroneously diagnosed bezoars as short SBFS in the intestinal loops. In cases like this, since the patient was unable to recognize dried persimmons as persimmons, patient questionnaires that mention dried persimmons may be useful in countries such as Japan, where there is a custom of eating them.

Third, prolonged conservative treatment can cause severe complications such as perforation. The administration of Coca-Cola is considered the primary choice for phytobezoar treatment because it is safe, inexpensive, and effective.^2)^ However, persimmon phytobezoars are characterized by their hard consistency and are often resistant to Coca-Cola dissolution, so clinicians may consider other types of treatments. Tannin and shibuol found in persimmons react with hydrochloric acid in the stomach and form a coagulum, which is the basis of a bezoar that accumulates cellulose, hemicellulose, and protein. In a systematic review, Ladas et al.^9)^ reported that only 23% of diospyrobezoars were dissolved after an initial attempt with Coca-Cola, while 60.6% of other phytobezoars were dissolved (*P* = 0.022). Coca-Cola is administered as a drinking beverage, as lavage, or as a combination of the two. Ladas et al.^9)^ reported that the volume of Coca-Cola used varied among studies on phytobezoars, and that the daily dose and duration of treatment varied from 500 to 3000 mL and from 24h to 6 weeks, respectively. Thus, no standard protocol for bezoar treatment has been established to date. Several authors have reported cases of intestinal obstruction and perforation resulting from diospyrobezoars.^13–15)^ Major complications of diospyrobezoars located in the small bowel include gastrointestinal bleeding due to ischemic ulceration of the intestinal mucosa and intestinal perforation with peritonitis.^15)^ In case reports, perforation cases show signs of peritoneal irritation, but this was not observed in this case. Suzuki et al.^14)^ also reported a case of small bowel perforation with preoperative fever only, without signs of peritoneal irritation. Therefore, in cases where conservative therapy is adopted, if fever or elevated CRP is observed, the possibility of perforation should be suspected, and surgery should be considered. Clinicians should recognize and pay attention to the occurrence of severe complications during Coca-Cola dissolution therapy.

## CONCLUSIONS

We experienced a case of bezoars-induced intestinal obstruction caused by dried persimmons that required reoperation. We learned 3 lessons: since persimmon bezoars may be present in more than 1 location, a search from the stomach to the small intestine is necessary in addition to the site of the obstruction. A detailed interview, including gathering information about dried persimmons, is necessary, and during conservative therapy, attention should be paid to the possible occurrence of severe complications.

## DECLARATIONS

### Availability of data and materials

The data that support the findings of this study are not publicly available due to patient privacy or ethical restrictions but are available from the corresponding author on reasonable request.
